# Plasma lipidomics analysis finds long chain cholesteryl esters to be associated with Alzheimer's disease

**DOI:** 10.1038/tp.2014.127

**Published:** 2015-01-13

**Authors:** P Proitsi, M Kim, L Whiley, M Pritchard, R Leung, H Soininen, I Kloszewska, P Mecocci, M Tsolaki, B Vellas, P Sham, S Lovestone, J F Powell, R J B Dobson, C Legido-Quigley

**Affiliations:** 1 Institute of Psychiatry, Psychology and Neuroscience, King's College London, London, UK; 2 Institute of Pharmaceutical Science, King's College London, London, UK; 3 Department of Neurology, Kuopio University Hospital and University of Eastern Finland, Kuopio, Finland; 4 Department of Old Age Psychiatry & Psychotic Disorders, Medical University of Łódź, Łódź, Poland; 5 Section of Gerontology and Geriatrics, Department of Medicine, University of Perugia, Perugia, Italy; 6 Memory and Dementia Center, 3rd Department of Neurology, Aristotle University of Thessaloniki, Thessaloniki, Greece; 7 Department of Internal and Geriatrics Medicine, INSERM U 1027, Gerontopole, Hôpitaux de Toulouse, Toulouse, France; 8 Department of Psychiatry, State Key Laboratory of Brain and Cognitive Sciences, and Centre for Genomic Sciences, Li Ka Shing Faculty of Medicine, the University of Hong Kong, Hong Kong; 9 Department of Psychiatry, University of Oxford, Warneford Hospital, Oxford, UK; 10 National Institute for Health Research Biomedical Research Centre for Mental Health and Biomedical Research Unit for Dementia at South London and Maudsley NHS Foundation, London, UK

## Abstract

There is an urgent need for the identification of Alzheimer's disease (AD) biomarkers. Studies have now suggested the promising use of associations with blood metabolites as functional intermediate phenotypes in biomedical and pharmaceutical research. The aim of this study was to use lipidomics to identify a battery of plasma metabolite molecules that could predict AD patients from controls. We performed a comprehensive untargeted lipidomic analysis, using ultra-performance liquid chromatography/mass spectrometry on plasma samples from 35 AD patients, 40 elderly controls and 48 individuals with mild cognitive impairment (MCI) and used multivariate analysis methods to identify metabolites associated with AD status. A combination of 10 metabolites could discriminate AD patients from controls with 79.2% accuracy (81.8% sensitivity, 76.9% specificity and an area under curve of 0.792) in a novel test set. Six of the metabolites were identified as long chain cholesteryl esters (ChEs) and were reduced in AD (ChE 32:0, odds ratio (OR)=0.237, 95% confidence interval (CI)=0.10–0.48, *P*=4.19E−04; ChE 34:0, OR=0.152, 95% CI=0.05–0.37, *P*=2.90E−04; ChE 34:6, OR=0.126, 95% CI=0.03–0.35, *P*=5.40E−04; ChE 32:4, OR=0.056, 95% CI=0.01–0.24, *P*=6.56E−04 and ChE 33:6, OR=0.205, 95% CI=0.06–0.50, *P*=2.21E−03, per (log2) metabolite unit). The levels of these metabolites followed the trend control>MCI>AD. We, additionally, found no association between cholesterol, the precursor of ChE and AD. This study identified new ChE molecules, involved in cholesterol metabolism, implicated in AD, which may help identify new therapeutic targets; although, these findings need to be replicated in larger well-phenotyped cohorts.

## Introduction

A better understanding of the biological mechanisms underlying Alzheimer's disease (AD) is required. AD is a devastating illness that currently affects over 496,000 people in the UK (www.alz.org.uk). It is one of the major challenges for health care in the 21st century and with estimated longer life expectancy, the number of demented patients worldwide are expected to reach 81.1 million in 2040.^1^ The lack of current treatments for AD and the lack of a definite and early diagnosis highlights the absence of a comprehensive understanding of the biological mechanisms underlying the changes that occur during the process of neurodegeneration. This underscores the urgent need for biomarkers that would lead to novel treatment strategies and improve the lives of those affected.^2^ Current sulcal cerebrospinal fluid or brain imaging biomarkers are costly, can cause discomfort to the patient and are impractical at large scale. For example, the Medicare Evidence Development and Coverage Advisory Committee concluded that there was too little evidence to show that amyloid positron emission tomography (PET) scans improve AD outcomes or that its benefits outweigh the harms and high cost.^3^ A blood-based biomarker could act as a screening tool to identify at-risk individuals for further investigation or recruitment into clinical trials.

Studies have now demonstrated the potential of using blood metabolites, the repertoire of molecules (size <1000–1500 Da)^4^ present in cells and tissue,^5^ as functional intermediate phenotypes in biomedical and pharmaceutical research.^6^ Metabolic phenotyping and its subset lipidomics is the rapidly evolving field of the comprehensive measurement, in a non-targeted manner, of ideally all endogenous metabolites in a biological sample.^7^ These small-to-medium molecules are the final products of interactions between gene expression, protein expression and the cellular and external environment, and represent an essential aspect of the phenotype of an organism.^8^ In addition, it is now known that there is a communication between the brain and the periphery and is increasingly believed that the damage to the blood–brain barrier caused by AD increases theoretically the chance of metabolites crossing to the brain.^2, 9, 10^ Coupled with the fact that blood is relatively easily accessible, plasma metabolites are an ideal source of noninvasive biomarkers and a molecular ‘footprint' of disease.

Recently, a number of non-targeted blood metabolomic studies (liquid chromatography-mass spectrometry (LC-MS) or direct infusion mass spectrometry (MS)) in AD have emerged, highlighting the role of lipid compounds, such as sphingolipids,^11^ bile acids,^12^ desmosterol^13^ and phosphatidylcholines (PCs).^14, 15, 16^ In a LC-MS/NMR screen followed by subsequent quantification, we previously identified three lipid PC molecules (PC16:0/20:5, PC16:0/22:6 and 18:0/22:6) that were progressively diminished in subjects with mild cognitive impairment (MCI) and AD patients.^16^ Interestingly, the latter two were found to be markers in a recent study where a panel of 10 lipids showed 90% area under curve (AUC) for preconversion to amnestic MCI or AD.^14^

Lipid metabolism has been extensively implicated in the pathogenesis of AD through cell biological^17, 18^ and genetic studies.^19, 20, 21, 22^ Further support comes from brain tissue metabolomic studies, which have shown changes in lipid compounds such as lysobisphosphatidic acid, sphingomyelin, sphingolipids and desmosterol in the brains of AD patients.^23, 24, 25, 26^

In this study, we performed untargeted lipidomics utilizing plasma samples from 36 AD patients, 40 healthy controls and 48 individuals with MCI with the aim of discovering new molecules that could diagnose AD patients from controls and MCI and improve our current knowledge of molecules associated with AD and their underlying biology.

## Materials and methods

### Patient sample collection

This study utilized 124 age and gender matched plasma samples (36 AD patients, 48 MCI individuals and 40 controls) from the Dementia Case Register (DCR) at King's College London and the EU funded AddNeuroMed study.^16, 27^ Normal elderly control subjects were recruited from non-related family members of AD patients, care-givers' relatives, social centres for the elderly or GP surgeries and had no evidence of cognitive impairment. AD and MCI subjects were recruited primarily from local memory clinics, and as such the MCI cohort was expected to be composed largely of subjects with a likely AD end point. Overlapping samples from this study were previously used by Whiley *et al.*^16^ to measure the relative amounts of three PCs to a PC internal standard in plasma. Relevant ethics board approved the study and informed consent was obtained for all subjects. Each patient was required to fast for 2 h before sample collection and 10 ml of blood was then collected in tubes coated with sodium ethylenediaminetetraacetic acid to prevent clotting. Whole blood was centrifuged to form a plasma supernatant, which in turn was removed and placed at −80 °C until further use.

### Lipidomics

#### Sample treatment

Sample treatment has been described elsewhere,^4,16^ 20 μl of plasma was added to a glass HPLC vial containing a 400 μl glass insert (Chromacol, Welwyn Garden City, UK). Ten microlitres of high purity water and 40 μl of MS-grade methanol were added to each sample, followed by a 2 min vortex mix to precipitate proteins. Then, 200 μl of methyl *t*-butyl ether (containing 10 μg ml^−1^ of internal standard triacylglycerol 45:0) was added, and the samples were mixed via vortex at room temperature for 1 h. After the addition of 50 μl of high purity water, a final sample mixing was performed before centrifugation at 3000 *g* for 10 min. The upper, lipid-containing, methyl *t*-butyl ether phase was then injected onto the LC-MS system directly from the vial by adjustment of the instrument needle height (17.5 mm from bottom).

The LC-MS-MS method employed has previously been published^2, 16^ and has shown to measure amounts of >4500 metabolite species, particularly lipids. Instrumentation included a Waters ACQUITY UPLC and XEVO QTOF system (Waters, Milford, MA, USA).

Chromatographic separation was achieved using an Agilent (Palo Alto, CA, USA) Poroshell 120 EC-C8 column (150 mm × 2.1 mm, 2.7 μm), maintained at 60 °C. A gradient was used consisting of 10 mM ammonium formate in water (A) and 10 mM ammonium formate in methanol (B). The solvent was delivered at a flow rate of 0.5 ml min^−1^. The gradient consisted of 0 min (75% B), 23 min (96% B), 36 min (96% B), 36.5 min (100% B), 41.5 min (100% B), 42 min (75% B), 51 min (75% B).

The XEVO QTOF was operated in the positive ion mode with a capillary voltage of 2.5 kV and a cone voltage of 60 V. The desolvation gas flow was 500 l h^−1^ and the source temperature was 120 °C. All analyses were acquired using the lock spray setting; leucine enkephalin was used as lock mass (*m*/*z* 556.2771 and 278.1141). Data were collected in the centroid mode over the mass range *m*/*z* 100–1000 with an acquisition time of 1 s per scan.

Samples were analysed in a randomized order, in four batches, with pooled plasma sampled (quality control (QC) samples) at regular intervals throughout the run (*n*=20). LC-MS raw data were aligned and normalized to total mean area, using Waters MarkerLynx software (Waters). Further validation of metabolite concentrations, which were associated with disease status took place using Waters QuanLynx software (Waters) and calculating peak ratios for metabolites that were over the LOQ (limit of quantification) and the area under the peak of the internal standard.

### Statistical analysis

#### Data pre-treatment

Metabolites detected in <80% of each of the diagnostic groups and the four batches were excluded from analyses. Metabolite distributions were inspected using histograms and the Shapiro–Wilks test was used to test for normality of the metabolite distributions. The distribution of a large number of metabolites was skewed and the data were further log2 transformed. The Empirical Bayes method ComBat^28^ was used to correct for batch effects in each metabolite. Principal components analysis was then used for the detection of outliers and to check whether the 20 QC samples clustered together. Missing data points were imputed using KNN (*k*-nearest neighbours, *k*=10), separately for each disease phenotype (‘impute'). Metabolite correlations were visualized using a heatmap (‘ggplot2'). Analysis of variance was used to test for differences in the levels of continuous variables between the three diagnostic groups, followed by Tukey's honest significant differences *post hoc* test between pairs of groups when the results of analysis of variance were significant at the *P*<0.05 level. Pearson's *χ*^2^ was used to test for differences in frequency of the categorical variables between the diagnostic groups. All the analyses took place in R.3.01.

#### Single analyte analysis

Analytes were centred around their mean. Logistic regression (‘glm') was used to investigate the association of each metabolite individually with disease status (AD versus controls). Logistic regression models were adjusted for the number of APOE ɛ4 alleles, age, gender and batch. False discovery rate (FDR) correction (0.05) was applied to correct for multiple testing. Secondary models were run to test whether the association of metabolites with disease status was modified by the presence of the APOE ɛ4 allele by testing for interactions.

#### Multivariate analysis

A Random Forest approach (using ‘rf' and ‘rfe' in the package ‘CARET') was used to develop an AD versus control classifier. Due to the large number of variables and their highly correlated structure, the following approach was used to achieve high performance with a minimal variable set. AD cases and controls were divided into a training (2/3) and an independent test data set (1/3) such that the training set comprised equal numbers of each diagnostic group. The training set was further divided into 100 bootstrap sample sets comprising a bootstrap training set (75%) used to build the Random Forest model and a bootstrap test set (25%) used to test the model. In each model, the default setting for ntree=500 was used and the optimum mtry number after 100 bootstraps was chosen and fitted to the whole training data set. The ‘AUC' was used to test for the performance of each classifier.

In each of the 100 bootstrap iterations, each variable was assigned a variable importance score. The ranks were summed for each metabolite over the 100 bootstraps providing an indication of the predictive power of each variable. We then selected the top 10% analytes on the basis of their summed variable importance rankings and ran the Random Forest with recursive feature elimination (rfe), that is, backward elimination on these selected variables using a second round of 100 bootstraps. The boostrapping was repeated keeping the top 50 to 10 analytes in steps of five, and down to two analytes in steps of one. For each subset of predictors, the mean bootstrap testing performance was calculated, and, on the basis of this, the optimal number of variables was identified using the ‘sizeTolerance' function. This takes into account the whole profile during ‘rfe' and picks a subset size that is small without sacrificing too much performance. The optimal number of variables was then used to build a final model in the complete training data, which was tested with the independent test set (Model 1).

The final model was also tested on the MCI sample to determine whether it would classify MCI as cases or controls.

We then repeated the steps above including APOE ɛ4 genotype (0,1 or 2 alleles) during the initial Random Forest bootstrapping step to assess its variable importance and the predictive ability of the metabolites in the presence of APOE.

## Results

The sample demographics are displayed in Table 1 and Supplementary Figure 1 gives a detailed account of the QC steps. In brief, 1878 molecular features were extracted from the 124 samples. Following QC, metabolites detected in <80% of the three diagnostic groups and the four batches were removed and 573 features were left for analysis. The distribution of >90% of the 573 features deviated from the normal, reflecting in some cases different batch distributions. The features therefore underwent log2 transformation and batch effect correction, resulting in >95% metabolites following a normal distribution. Principal components analysis showed that all 20 QC samples clustered together (Supplementary Figure 2), verifying reproducible results across the batches and highlighting the presence of one outlier (AD case), which was removed. KNN imputation was performed on the 123 samples with the 573 features.

### Single analyte analyses results

Logistic regression analyses for AD versus controls, adjusting for the number of APOE ɛ4 alleles, age, gender and batch, indicated that 95 analytes were associated with AD at *P*-value <0.05 and 41 analytes at *q*-value <0.05. Results for all 573 analytes and all pair-wise comparisons are provided in Supplementary Table 1. Secondary models investigating for APOE ɛ4 specific associations by testing for interactions between analytes and the APOE ɛ4 allele indicated that the association of 29 analytes with AD seemed to be modified for the APOE ɛ4 at *P*-value <0.05; However, none of these associations were significant at *q*-value <0.05 (results not presented).

### Multivariate analysis results

Random Forest was performed on the training data set using the 573 features (100 bootstraps). Supplementary Figure 3 displays the variable importance after 100 bootstraps, with the dotted line showing a slight leveling off in the importance measure and which corresponds to the top 10% features (*n*=57). Random Forest with ‘rfe' on the training data set showed that the highest mean training performance was for a model with 25 features. However, to choose a model with high accuracy while reducing the number of features as low as possible, a model with 10 molecules was chosen (AUC=0.867) (Supplementary Figure 4). We fitted the Random Forest model with the 10 selected variables on the whole training data set, which predicted the training data set with 82.35% accuracy (sensitivity=87.5%, specificity=77.8%, AUC=0.826) and could classify our test data set with 79.2% accuracy, 81.8% sensitivity, 76.9% specificity, a positive predictive value of 75.0%, a negative predictive value of 83.3% and an AUC of 0.792.

When we repeated the analysis including APOE, APOE had very low variable importance (222 out of 573) and was not selected forward. In addition, APOE was not selected in the final model after forcing it during ‘rfe' together with the top 10% of the selected analytes from the 100 Random Forest bootstraps.

**Figure 1:**
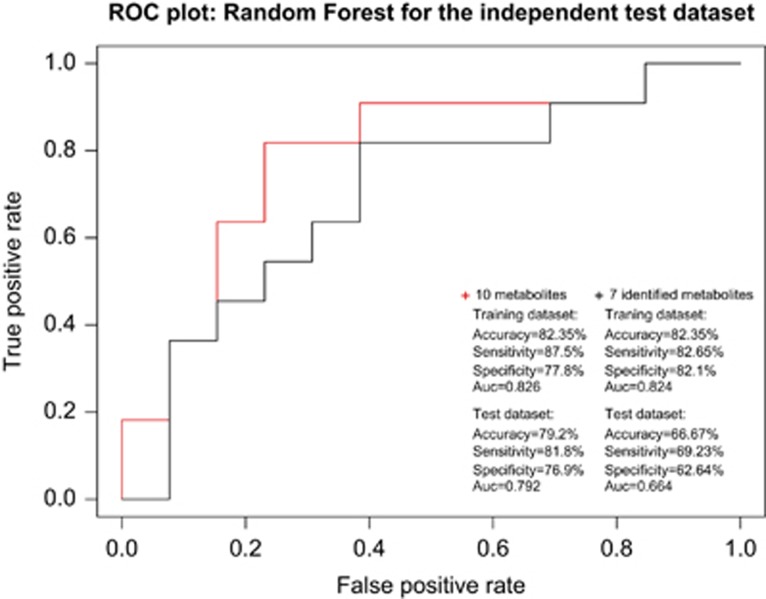
Receiver operator curve (ROC) for the independent test data set for the selected 10 metabolites after recursive feature elimination for AD versus controls and for the seven identified metabolites, and summaries of the classifier models for the training and test data sets. AD, Alzheimer's disease; AUC, area under curve.

Results for the Random Forest model are displayed in Figure 1. Nine of the 10 metabolites of the classifier were associated with AD in single analyte regression analysis at *q*<0.05 (Supplementary Table 1 and Table 2) and none of them was shown to interact with the APOE ɛ4 genotype at *P*<0.05.

### Identification and measurement of putative biomarkers

**Figure 2:**
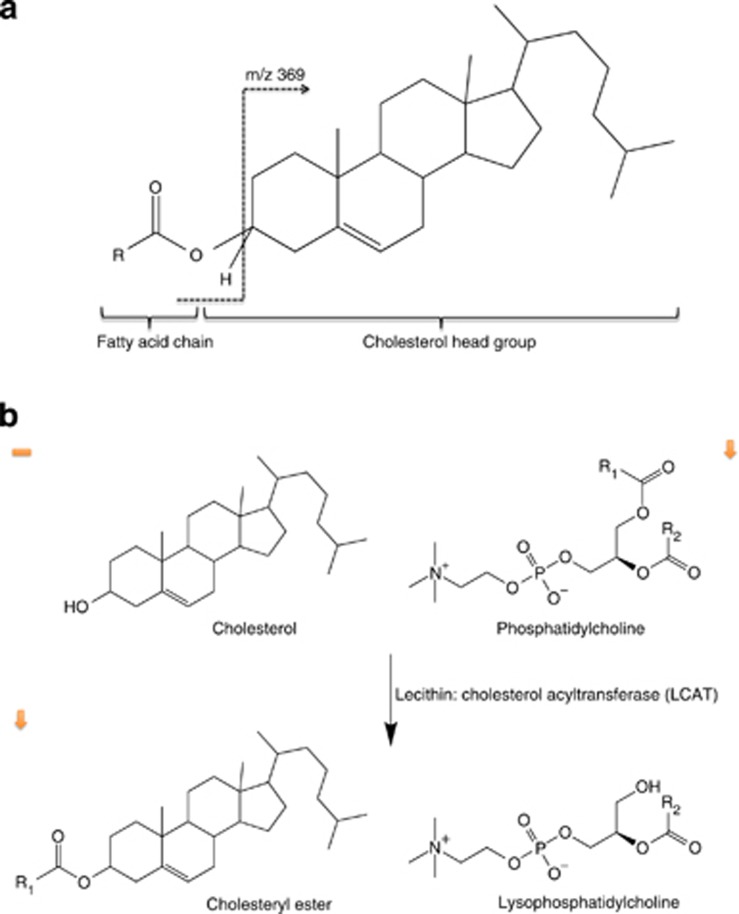
Cholesteryl ester molecules structure (**a**) and synthesis from cholesterol catalysed by lecithin: cholesterol acyl transferase (LCAT) (**b**). ChE is characterized by the presence of cholesterol head group in ESI(+) of 369.

Six molecules were identified using MS/MS fragmentation patterns (Figure 2a) and found to be cholesteryl esters (ChEs) with very long chain fatty acids,^29, 30^ synthesized from cholesterol (Figure 2b). These were *m*/*z* 866 (ChE 32:0), *m*/*z* 894 (ChE 34:0), *m*/*z* 882 (ChE 34:6), *m*/*z* 856 (ChE 32:4), *m*/*z* 868 (ChE 33:6) and *m*/*z* 970 (ChE 40:4). In addition, one molecule was proposed as an oxidized form of desmosterol following the expected fragmentation pattern (*m*/*z* 367). The six identified ChE molecules and the desmosterol-related molecule predicted the training data set with 82.35% accuracy (sensitivity=82.61%, specificity=82.14%, AUC=0.824) and could classify the test data set with 67% accuracy, 69.23% sensitivity, 62.64% specificity, a positive predictive value of 69.23%, a negative predictive value of 63.64% and an AUC of 0.670.

Relative quantification was produced for eight of the molecules, which were consistently over the limit of quantification (LOQ). Univariate association analysis of the eight peak ratios revealed that the associations of all of them were strengthened. We, additionally, measured cholesterol, the precursor of ChE, finding no association with AD (Table 2).

**Figure 3:**
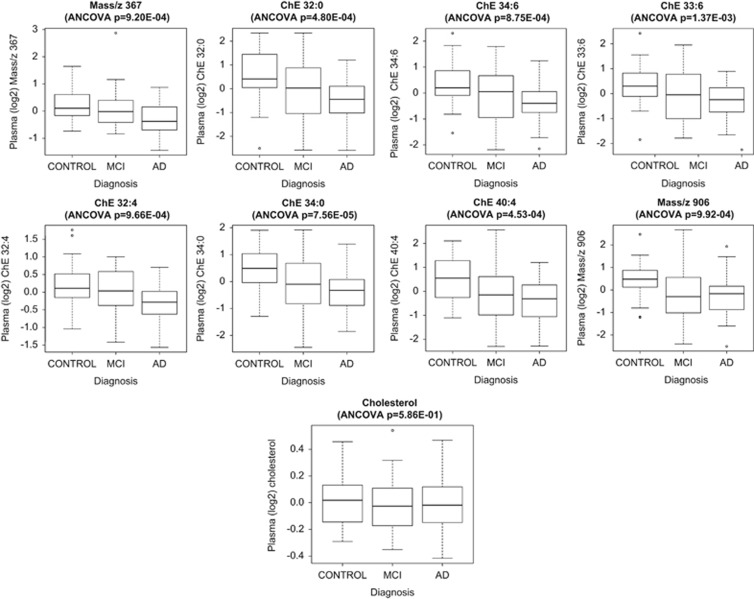
Boxplots depicting change in the level of the Random Forest measured molecules which were consistently above the level of quantification (LOQ) and that of cholesterol in the three diagnostic groups. All molecules were decreased in AD compared with controls after putative biomarker measurement. A decrease is also observed in MCI compared with controls. The ANCOVA *P*-value for the difference in metabolite levels in the three groups is displayed above each graph. AD, Alzheimer's disease; ANCOVA, analysis of covariance; MCI, mild cognitive impairment.

Figure 3 plots the levels of the metabolites and cholesterol in the three diagnostic groups, which highlights the overall decrease pattern in metabolite levels from healthy controls to MCI to AD. To further investigate the association of the metabolites with cognition, we investigated their correlation with MMSE, which showed a weak association (Supplementary Figure 5).

When we tested our classifier on individuals with MCI, 22 were classified as AD and 18 as controls.

Finally, logistic regression analyses including statin use and smoking status as covariates produced identical results (not presented).

## Discussion

We performed lipidomics on plasma samples from 36 late-onset AD patients, 40 healthy controls and 48 individuals with MCI to identify metabolites that differentiate between AD patients and healthy controls. Univariate analysis identified 41 analytes associated with AD (*q*<0.05). We then applied Random Forest and a backwards elimination approach to identify a reproducible metabolic signature to classify AD patients and we found a combination of 10 metabolite molecules that classified AD patients in the independent test data set with 79% accuracy. This is one of the largest non-targeted plasma studies to date to use such a systematic analysis pipeline, which included assessment of the model in an independent data set (1/3 of sample), to identify new targets linked to AD and provide improvement to current diagnostic classifiers.^11, 12, 13, 14, 15, 16^ Although APOE was associated with AD in single analyte analyses, its variable importance in the presence of the metabolites during classification was low and was not included in the classifier probably due to the metabolites already capturing the information that APOE provides. It should be noted that the single metabolite logistic regression results indicated that the metabolites provided information over and above APOE.

Most of the metabolites in the classifier were reduced in AD patients. We observed that their levels in MCI subjects were more variable but overall followed the control>MCI>AD trend with some of them being different at *P*<0.05 between AD and MCI or MCI and controls. To investigate this further, we plotted the correlation of metabolites with MMSE, which is a more informative continuous surrogate marker for disease status and observed weak association between the metabolic signature and cognition.

We subsequently extracted single metabolite measures and observed that their associations with AD were strengthened (Table 2). Six molecules were identified as ChEs, molecules which have not been previously associated with AD and one as a potentially oxidized form of desmosterol, which could predict the test data set with 67% accuracy highlighting both their good predictive value and also the importance of the unidentified molecules in AD. We, additionally, measured cholesterol, as it is the precursor of ChE, and wanted to explore the chemical path differences with AD, finding no association. This suggested that the association with AD is specific to metabolites synthesized from cholesterol rather than to cholesterol itself.

**Figure 4:**
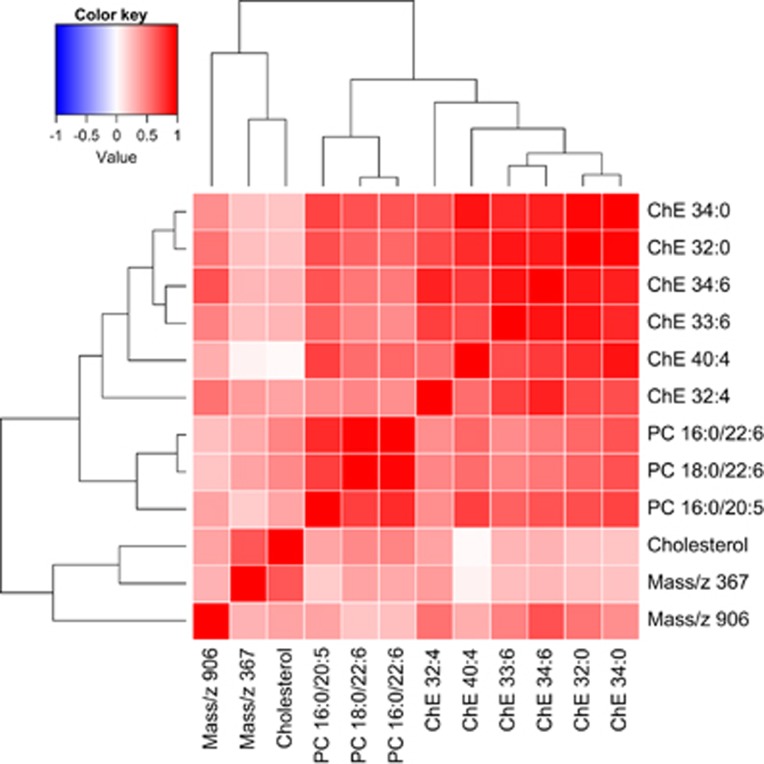
Heatmap of the eight measured metabolites selected during Random Forest classification, following recursive feature elimination, which were consistently above the level of quantification (LOQ). Cholesterol and the three phosphatidylcholines (PCs) previously published by Whiley *et al.*^16^ are also included. AD, Alzheimer's disease; ANCOVA, analysis of covariance; MCI, mild cognitive impairment.

A heatmap of the measured molecules together with cholesterol is shown in Figure 4. We have previously reported three PCs to be decreased in AD versus controls^16^ using overlapping samples. Although these PCs were not included in our classifier, their raw values were decreased in our data set (*P*<0.05) with one of them (mass 780 which is PC16:0/20:5) being associated with AD at *q*<0.05. These molecules were also recently identified as markers of phenoconversion to either amnestic MCI or AD.^14^ We have therefore included these three PCs in the heatmap to compare their biological variation with the newly discovered and biochemically related ChEs, which highlights the positive correlation between the metabolites identified here, cholesterol and the three PCs; therefore, it is likely that the information the PCs provide is captured by the analytes selected in the rfe step.

### Role of cholesterol and its derivatives

Cholesterol esters are largely synthesized in plasma by the transfer of fatty acids to cholesterol from PC, a reaction catalysed by the enzyme LCAT (lecithin: cholesterol acyl transferase). Free cholesterol can be taken up by APOE-containing lipoproteins, such as HDL, but is confined to the outer surface of the particle. The esterification of cholesterol to ChE ensures that more cholesterol is packaged into the interior of lipoproteins and this increases their capacity, allowing more efficient cholesterol transport through the bloodstream. LCAT has a preference for plasma 16:0–18:2 or 18:0–18:2 PCs, therefore, connecting the PCs identified by Whiley *et al.*^16^ and the ChEs identified here through a one-step enzymatic reaction (Figure 2b). Interestingly, LCAT is also expressed in the brain by astrocytes, and, together with APOE and ABCA1, has a key role in the maturation of glial-derived nascent lipoproteins.

In other tissues, cholesterol is esterified by acyl-coenzyme A: cholesterol acyltransferase 1 and 2 (ACAT1 and ACAT2). ACAT uses acyl-CoA as a source for the acyl chains. Recent evidence suggests a strong link of ACAT1 with amyloid deposition. Pharmacological inhibition of ACAT in AD mice resulted in diminished amyloid plaque burden in their brains and improved cognitive function^25, 31^ and genetic ablation of Acat1 in AD mice diminished the levels of Aβ42, decreased the amyloid plaque burden and full-length human APP (human amyloid precursor protein), and improved the cognitive function of the mice.^32^ Finally, a recent gene therapy study showed that adeno-associated virus targeting Acat1 for gene knockdown delivered to the brains of AD mice decreased the levels of total brain amyloid-β, oligomeric amyloid-β and full-length human APP to levels similar to complete genetic knockdown of Acat1.^33^

Approximately one-third of the ChE is transferred from HDL to APOB-containing lipoproteins, such as VLDLs, in exchange for triglycerides by ChE transfer protein. Thus most of the cholesterol in circulating lipoproteins is ChE produced from HDL lipoproteins by the LCAT-catalysed reaction. This process results in lower HDL cholesterol and indirectly decreases HDL size as frequently observed in type 2 diabetes^34^ and a deficiency of ChE transfer protein is associated with increased HDL and decreased LDL levels, a profile typically antiatherogenic.^35, 36^ Interestingly, an elevation of cholesterol esters (chains 14:20) was observed in the brains of AD patients and of three transgenic familial AD mouse models.^24^

Lower desmosterol levels have been previously found in the plasma and brains of AD patients compared with healthy controls.^13, 26^ Desmosterol is a precursor of cholesterol and seladin (*DHCR24*), which governs the metabolism of desmosterol to cholesterol in specific brain areas has been shown to counteract the β-secretase cleavage of APP and the formation of amyloid-β.^37, 38^ An unknown molecule with similar structure and the same mass as desmosterol shows the highest correlation with cholesterol here.

### Strengths and limitations

To our knowledge, ours is the first study to implicate ChEs in AD. We should highlight, however, that these molecules are biochemically related to metabolites previously associated with AD initiation and progression and are, therefore, biologically relevant. Although lipid molecules have been previously implicated in AD, results are not always consistent. It is worth noting that even if previously published studies go under the umbrella of plasma lipidomics and/or metabolomics, differences are considerable in terms of patient cohort selection (such as differences in patient groups selection, in age and in dementia severity), in terms of technical treatment (such as sampling/storage conditions and fasting state before sample collection) and in clinical data availability. As an example, sample groups can differ from a simple case versus control design^11^ to including MCI^12, 13, 14, 15, 16^ and in one case including time points with pre- and post-conversion patients.^14^ Sample groups will influence the selectivity of results, as well as patient numbers, which ranged from <50 in early studies^11^ to the low hundreds in more recent studies.^13, 14, 15, 16^ In addition, LC-MS fingerprinting methods can be qualitative or/and quantitative, hence the information can be relative amounts^11, 12, 15^ or a combination of relative and absolute amounts for chosen metabolites.^13, 14, 16^ Another factor for differences observed between studies would be the different data processing tools applied for data mining and statistical methods used to produce AD biomarker predictive models. Some studies showed predictive models of AD based on one to three molecules^13, 16^ or a battery ranging from three to 10 markers and have used a wide range of statistical analysis methods ranging from simple single metabolite approaches^11^ to methods using multivariate regression approaches and resampling techniques,^15^ and validation of the panels in independent data sets.^14^ Here, we have used a well-characterized AD cohort matched for age and gender and performed a careful and systematic analysis pipeline to extract metabolites associated with AD, by performing bootstrapping to avoid over-fitting and validating our results in an unseen data set. After the molecules associated with AD were identified, an additional method of metabolite quantification in all the samples was performed, which was possible due to internal standards and a run separating lipids, which acquired data for 2 h for each sample.

Our study also suffers from potential limitations. Although ours is one of the largest AD metabolomics studies to date, the sample size is still modest and replication is required in larger cohorts. In addition, this study suffers from limitations inherent to AD case–control studies, such as the possibility that some of the healthy elderly controls may already carry pathology, and that some of the clinically diagnosed AD may be pathologically non-AD dementias. Through follow-up data on the individuals used in this study, however, we know that all AD patients used for our analysis maintained the diagnosis of AD as did all controls for at least 3 years from their baseline visit. We also believe that achieving such high performance in both training and test data sets for an AD case–control data set highlights the efficacy of the classifier, as AD diagnostic classifiers rarely achieve such high performance; we, therefore, believe that having additional pathology information would only increase its performance. Furthermore, we need to acknowledge that individuals with MCI are more heterogeneous in pathology; however, the MCI subjects used in this study were recruited primarily from local memory clinics and were, therefore, expected to be composed largely of subjects with likely an AD end point.

Finally, due to the large number of comorbidities in old age, we have to acknowledge that our metabolite signal may not be AD specific but it could it be associated with overall ill health and other comorbid conditions, making it potentially a not good biomarker for recruitment in clinical trials. Investigating and comparing the metabolic profiles of other disorders would increase the specificity of our panel.

## Conclusions

In this study, we used a Random Forest approach and identified a combination of 10 metabolites, which predicted AD with near 80% accuracy. We subsequently identified six of the metabolites to be ChEs, molecules not previously implicated in AD, which are connected to PCs through a one-step enzymatic reaction. The newly identified molecules were reduced in AD patients compared with controls. All these, combined with the lack of association between cholesterol and AD, suggest that it is the dysregulation of specific steps in cholesterol metabolism, rather than cholesterol itself, that is responsible for these observations and suggest novel targets for future work. These findings need to be replicated in larger well-phenotyped cohorts, which will be possible in the near future through the large biomarker consortia being set up. In addition, information on pathology status, such as amyloid marker cerebrospinal fluid, PET or brain atrophy measurements, through magnetic resonance imaging (MRI), would provide more precise phenotypes for biomarker discovery and would capture different stages of disease pathology, and, the comparison of metabolite levels between MCI patients who converted to AD and those who remained stable would provide us with metabolites, which are associated with disease initiation. Finally, integrating additional types of biological modalities, such as protein, gene expression and genotype information, will help investigate the origin of ChE dysregulation in AD.

## Figures and Tables

**Table 1:** Sample demographics

	*Comprehensive plasma LC-MS lipidomics (*n=*124)*			
	*Control*	*MCI*	*AD*	*ANOVA F(df1,df2) or x2(df) tests*
*N*	40	48	36	
Age (mean, s.d.)	78.46 (6.7)	78.96 (5.6)	78.14 (7.7)	F=0.180 (2,121), *P*=0.836
MMSE (mean, s.d.)	29.00 (1.1)	26.94 (1.9)	21.49 (4.8)	F=65.46(2,121), *P*<2.0E−16^a^
Female/male (*N*)	17/18	26/22	21/19	*χ*^2^=0.259 (2), *P*=0.879
APOE ɛ4 alleles (0/1/2) (*N*)	33/7/0	28/12/3	15/15/6	*χ*^2^=12.812 (2), *P*<16.5E−03^b^
Batch (1/2/3/4) (*N*)	9/10/9/12	14/11/10/13	9/8/10/9	*χ*^2^=1.256 (2), *P*=0.974
Diabetes^c^	1	3	3	*χ*^2^=0.749 (2), *P*=0.688
Smoking^d^	6	0	9	*χ*^2^=2.729 (2), *P*=0.255
Statins	10	20	14	*χ*^2^=3.552 (2), *P*=0.169
Samples (LON)	40	28	36	NA
Samples (EUR)	0	20	0	NA

Abbreviations: AD, Alzheimer's disease; ANOVA, analysis of variance; EUR, samples obtained from the non-London AddNeuroMed European centres; LC-MS, liquid chromatography-mass spectrometry; LDN, samples obtained from London AddNeuroMed- and DCR-based patients; MCI, mild cognitive impairment; MMSE, Mini-Mental State Examination score; NA, not available.

a Tukey's honest significant differences *post hoc* tests: AD versus Control *P*<1.0E−17; AD versus MCI *P*=4.02E−13; MCI versus Control *P*=3.36E−03.

b AD versus Control *P*=7.5E−04; AD versus MCI *P*=0.1364; MCI versus Control *P*=0.099.

c Diabetes information was available for 34 AD patients, 29 controls and 43 MCI.

d Information on smoking was available for 34 AD patients, 29 controls and 6 MCI.

**Table 2:** List of metabolite molecules selected by the Random Forest classifier (AD versus elderly health controls)

*Metabolite molecule*	*Peak height data normalized to whole mean only*			*Peak ratios normalized to internal standard*		
	*OR*	*95% CI*	P*-value*	*OR*	*L95 95% CI*	P*-value*
Mass/*z* 367	0.124	0.03–0.39	1.31E−03	0.115	0.03–0.36	8.09E−04
ChE 32:0	0.251	0.10–0.52	7.51E−04	0.237	0.10–0.48	4.19E−04
ChE 34:0	0.151	0.05–0.38	3.14E−04	0.152	0.05–0.37	2.90E−04
ChE 34:6	0.231	0.08–0.53	1.56E−03	0.126	0.03–0.35	5.40E−04
ChE 32:4	0.141	0.04–0.43	1.75E−03	0.056	0.01–0.24	6.56E−04
ChE 33:6	0.218	0.07–0.55	3.15E−03	0.205	0.06–0.50	2.21E−03
Mass/*z* 628	3.669	1.20–13.93	3.49E−02	NA	NA	NA
Mass/*z* 906	0.210	0.07–0.50	1.40E−03	0.226	0.09–0.48	3.73E−04
Mass/*z* 315	5.084	1.78–16.88	4.12E−03	NA	NA	NA
ChE 40:4	0.362	0.18–0.67	2.56E−03	0.279	0.12–0.55	6.43E−04
Cholesterol	NA	NA	NA	0.316	0.02–5.01	4.21E−01

Abbreviations: AD, Alzheimer's disease; ChE, cholesteryl ester; CI, confidence interval; NA, not available; OR, odds ratio.

Results are presented for peak height and peak ratio (measurement normalized to internal standard) after covariate adjustment. The metabolites are ordered according to their importance during recursive feature elimination. All metabolites except for mass 628 were associated with AD in single analyte regression analysis (*q*<0.05). Results for semiquantified mass 628 and mass 315 are not presented as they were not consistently above the limit of quantification. Results are also presented for the APOE ɛ4 allele and for cholesterol peak ratio. The association of APOE ɛ4 was OR=5.620, 95% CI=2.27–16.29, *P*=5.48E−04 per ɛ4 allele.
