# Supplementary material for: Plasma lipidomics analysis finds long chain cholesteryl esters to be associated with Alzheimer's disease
Source: Transl Psychiatry. 2015 Jan 13;5(1):e494–. doi: 10.1038/tp.2014.127 (PMC4312824; doi:10.1038/tp.2014.127)
Supplement: Supplementary Figures [file tp2014127x2.doc]

Supplementary Figure 1. Quality control steps and analysis pipeline employed in this study.


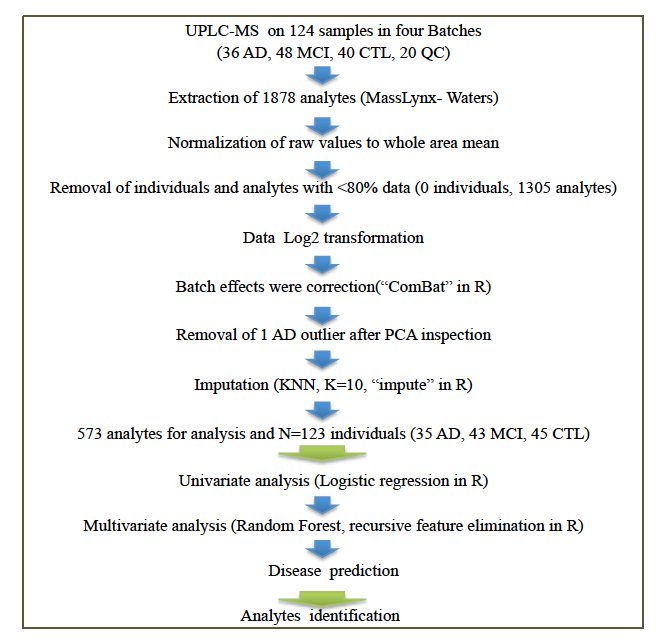


Supplementary Figure 2. PCA plot of the log2 transformed and batch corrected data showing the clustering of the 20 QC samples from the four experimental batches.


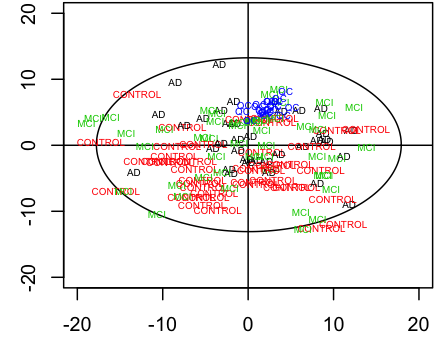


Supplementary Figure 3. Ranking of the 573 metabolites after Random Forest on the training dataset according to their variable importance (VI) after 100 Bootstraps. A slight leveling off in the importance measure corresponded to the top 10% features (n=57).

Supplementary Figure 4. Receiver operator curve (ROC) values for the recursive feature elimination (rfe) on the top 100 metabolites using the Random Forest function and a decreasing number of variables in each step.

The ROC value is the average ROC value after 100 Bootstraps for each combination of variables. The highest ROC value was reached when 25 metabolites were used in the model. However we achieved similar ROC value without sacrificing accuracy when ten metabolites were used.

Supplementary Figure 5. Scatterplots depicting the correlation between MMSE taken at the visit as the plasma visit with levels of the ten measured metabolites and cholesterol. The black line plots the correlation of metabolites with MMSE for the whole sample.
